# Genome-wide identification of *WRKY* family genes in peach and analysis of *WRKY* expression during bud dormancy

**DOI:** 10.1007/s00438-016-1171-6

**Published:** 2016-03-07

**Authors:** Min Chen, Qiuping Tan, Mingyue Sun, Dongmei Li, Xiling Fu, Xiude Chen, Wei Xiao, Ling Li, Dongsheng Gao

**Affiliations:** College of Horticulture Science and Engineering, Shandong Agricultural University, 61 Daizong Road, Tai’an, 271018 China; State Key Laboratory of Crop Biology, Shandong Agricultural University, 61 Daizong Road, Tai’an, 271018 China; Shandong Collaborative Innovation Center for Fruit and Vegetable Production with High Quality and Efficiency, 61 Daizong Road, Tai’an, 271018 China

**Keywords:** WRKY transcription factors, Peach, Bud dormancy

## Abstract

Bud dormancy in deciduous fruit trees is an important adaptive mechanism for their survival in cold climates. The *WRKY* genes participate in several developmental and physiological processes, including dormancy. However, the dormancy mechanisms of *WRKY* genes have not been studied in detail. We conducted a genome-wide analysis and identified 58 *WRKY* genes in peach. These putative genes were located on all eight chromosomes. In bioinformatics analyses, we compared the sequences of *WRKY* genes from peach, rice, and *Arabidopsis*. In a cluster analysis, the gene sequences formed three groups, of which group II was further divided into five subgroups. Gene structure was highly conserved within each group, especially in groups IId and III. Gene expression analyses by qRT-PCR showed that *WRKY* genes showed different expression patterns in peach buds during dormancy. The mean expression levels of six *WRKY* genes (Prupe.6G286000, Prupe.1G393000, Prupe.1G114800, Prupe.1G071400, Prupe.2G185100, and Prupe.2G307400) increased during endodormancy and decreased during ecodormancy, indicating that these six *WRKY* genes may play a role in dormancy in a perennial fruit tree. This information will be useful for selecting fruit trees with desirable dormancy characteristics or for manipulating dormancy in genetic engineering programs.

**Electronic supplementary material:**

The online version of this article (doi:10.1007/s00438-016-1171-6) contains supplementary material, which is available to authorized users.

## Introduction

To endure harsh environmental conditions in winter, perennial deciduous fruit trees have developed adaptation mechanisms such as dormancy and overwintering (bud dormancy). In recent years, with global warming, perennial deciduous fruit trees have shown irregular phenologies because of inadequate winter chilling. These irregularities adversely affect fruit production (Luedeling et al. 2011). Thus, bud dormancy plays a critical role in the development of perennial plants, and research on bud dormancy is useful for the development of perennial deciduous fruit trees. There are several types of dormancy in plants; ecodormancy, paradormancy, and endodormancy (Lang 1987). Endormant buds cannot initiate growth under favorable conditions without prior chilling (Crabbe and Barnola 1996; Faust et al. 1997). Because endodormancy is irreversible, dormancy is one of the key factors limiting fruit production. The *WRKY* transcription factors (TFs) are involved in seed dormancy in *Arabidopsis*, but it is unknown whether they are involved in bud dormancy in perennial plants. Therefore, the identification of *WRKY* and dormancy-related *WRKY* genes in peach not only provides a molecular foundation for studies on bud dormancy, but will also be useful for exploring the functions of *WRKY* gene products.

Dormancy is an important state in which meristem activity ceases and the meristem is insensitive to growth-promoting signals (Rohde and Bhalerao 2007). The plant cannot resume growth until dormancy is released (Rohde and Bhalerao 2007). In bud dormancy, *FLOWERING LOCUS T* (*FT*) is a versatile regulator in the environmental control of meristem transitions including the cessation of growth and the establishment of dormancy (Henrik et al. 2006). In *Populus*, *FT* was rapidly induced by the short day-length signal (Henrik et al. 2006). In grapevine, *VvFT* was detected in leaves and buds under a short day-length photoperiod (Vergara et al. 2015), indicating that *FT* played a key role in regulating the cessation of growth and establishment of endodormancy. A natural mutant of peach (*Prunus persica*) that could not express six MICK-type *MADS* genes at the EVG locus was unable to cease growth and form buds. This observation suggested that *MADS* box genes may be major candidates for controlling growth cession (Bielenberg et al. 2004, 2008). Subsequently, *PpDAM1*, *PpDAM2*, and *PpDAM4* were shown to be closely related to terminal bud formation (Li et al. 2009) and the transcript abundances of *PpDAM5* and *PpDAM6* were inversely with the sprouting rate of terminal buds in peach (Jiménez et al. 2009). *PpDAMs* were shown to play roles not only in inducing endodormancy, but also in releasing endodormancy (Yamane et al. 2011). Recently, *SOC1* (encoding a MADS-domain TF) was shown to affect the duration of dormancy in kiwifruit (Voogd et al. 2015). In seed dormancy, *DELAY OF GERMINATION1* (*DOG1*) is a major regulator of dormancy. In *Arabidopsis*, the levels of the DOG1 protein, which were largely independent of abscisic acid (ABA), functioned as a timer for seed dormancy release in freshly harvested seeds (Nakabayashi et al. 2012). *SNL1* (*SIN3*-*LIKE1*) and *SNL2* were also shown to be related to seed dormancy via their role in mediating the ABA-ethylene antagonism in *Arabidopsis* (Wang et al. 2013). *AtWRKY41* was shown to control both primary seed dormancy and thermo inhibition via directly regulating *ABI3* expression in mature imbibed seeds (Ding et al. 2014). As we known, both types of dormancy are characterized by a temporary insensitivity to growth-promoting signals and may have similar molecular mechanisms (Fu et al. 2014). In Persian walnut, Vahdati et al. (2012) also confirmed a relationship between the two types of dormancy breaking mechanism. WRKY TFs involved in seed dormancy have been identified, and whether they have a relationship with bud dormancy is unknown. Thus, it is necessary to verify the hypothesis.

The WRKY family is one of the ten largest families of TFs. This family, which is predominant in plants, is considered to be plant specific. Members of the WRKY family play crucial roles in regulating plant growth and development. The name, WRKY, is derived from the conserved domain of a WRKYGQK hexapeptide sequence at the N-terminus. These TFs also have a novel zinc-finger-like motif at the C-terminus, and form a four-stranded β-sheet and a zinc-binding pocket in which zinc coordinates with Cys/His residues to form the WRKY domain (Rushton et al. 2010). Although research on WRKY TFs has rapidly expanded from model plants to crop species, our knowledge of WRKY TFs in fruit trees, including peach, is limited. Since the first isolation of a WRKY protein (*SPF1*) from sweet potato in 1994, many other WRKY TFs have been identified from various plants including parsley *Arabidopsis*, wild oat, tobacco, and cucumber (Ishiguro and Nakamura 1994; Rushton et al. 1995, 1996). Previous studies have shown that WRKY TFs bind to certain promoters containing a W box (TTGACC/T), a cognate *cis*-acting element. Early research on WRKY TFs suggested that their main roles were in responses to pathogens (Eulgem and Somssich 2007). For example, PopP2 and AvrRps4 were shown to interact with WRKY domain-containing proteins (e.g. NB-LRR proteins) in *Arabidopsis* (Sarris et al. 2015). Recently, however, WRKY TFs have been shown to function in diverse processes such as germination, dormancy, and responses to abiotic stresses (Rushton et al. 2010). Many studies have demonstrated that members of the WRKY family play complex and sometimes contradictory regulatory roles in biotic stress responses. For example, *OsWRKY45*-*1* and *OsWRKY45*-*2* were shown to play opposite roles in regulating resistance to *Xanthomonas oryzae,* but identical roles in regulating resistance to *Magnaporthe grisea* (Masaki et al. 2007; Tao et al. 2009). Several WRKY TFs have been implicated in seed development, such as *Arabidopsis AtWRKY10* and *Solanum chacoense ScWRKY1* (Sun et al. 2003; Lagacé and Matton 2004). In wild oat, *ABF1* and *ABF2* were shown to bind to W boxes in the promoters of genes encoding α-amylases, which are crucial for starch hydrolysis during germination in cereals. Thus, *ABF1* and *ABF2* were shown to affect germination, and indirectly, post-germination. *AtWRKY6* was implicated in the regulation of leaf senescence and was strongly up-regulated during senescence (Robatzek and Somssich 2001; Silke and Somssich 2002). In subsequent studies, *AtWRKY53*, *AtWRKY70,* and *OsWRKY23* were also shown to regulate senescence. Other studies have shown that WRKY TFs participate in multiple processes. For example, *HvWRKY38* and *HvWRKY1* were shown to provide a mechanistic link among biotic stress responses, germination, and abiotic stress responses. *OsWRKY53* acted as a negative feedback modulator of *MPK3* and *MPK6* (Hu et al. 2015). Cai et al. (2015) demonstrated that *CaWRKY6* activates *CaWRKY40*, which functions as a positive regulator of *Ralstonia solanacearum* resistance and heat tolerance. Few dormancy-related *WRKY* genes have been identified so far. Therefore, it is important to identify which *WRKY* genes, if any, are related to dormancy in perennial species. The full genome sequence of peach is now available in public databases. Therefore, the aim of this study was to identify the *WRKY* genes, and specifically the dormancy-related *WRKY* genes, in the peach genome.

We searched the recently released peach genome and identified 58 candidate *WRKY* genes, which were distributed on all eight chromosomes. The genes were classified into three main groups according to their predicted WRKY domains and zinc finger structures. Gene structure analyses showed that the structures were highly conserved within each group. Finally, we analyzed the expression profiles of the *WRKYs* in bud dormancy, and identified six *WRKY* genes that may play important roles in dormancy.

## Materials and methods

### Plant materials

Peach samples were obtained from the Horticulture Experimental Station of Shandong Agricultural University, Tai’an, China. The plant materials (‘*Prunus persica* L. cv Zhong You Tao 4’) were grown under standard agricultural practices for 5 years. Bud samples for this study were collected from at least 30 independent trees. For analyses of gene expression at different stages of bud dormancy, peach bud samples were collected before leaf abscission, during dormancy, and during the dormancy-release period, on 16 and 31 October, 15 November, 1, 15 and 31 December, 15 and 25 January, and 15 February. At each time point, flower buds (500 mg) with scales were collected from first-year branches of different vigorous individual trees, and then the samples were immediately frozen in liquid nitrogen. The samples were stored at −80 °C until use.

### Definition of bud dormancy

To evaluate bud dormancy, we used 120 first-year branches incubated in 5 % (w/v) sucrose solution each time. The branches were collected on 16 and 31 October, 15 November, 1, 15 and 31 December, 15 and 25 January, and 15 February, and were incubated in a growth chamber. Trials were conducted in a completely randomized design with three replicates, each with 40 cuttings. The branches were kept under a 16-h light/8-h dark photoperiod with artificial fluorescent light (200 μmol m^−2^ s^−1^) with day/night temperatures of 25/18 °C and 70 % relative humidity. The basal ends of the shoots were cut weekly, and the sucrose solution was replaced daily. Sprouting was recorded after 25 days and 50 % bud sprouting marked the beginning of dormancy release. The results are expressed as percentage of budbreak for the three replicates.

### Identification of *WRKY* genes in peach

To identify the members of the *WRKY* gene family in peach, we conducted BLASTP (http://blast.ncbi.nlm.nih.gov/Blast.cgi) searches using the proteome sequences as a database. Annotated peach WRKY proteins were used as query sequences to perform BLAST searches against the proteome and genome files downloaded from the peach genome database (https://www.rosaceae.org). To verify the authenticity of candidate sequences, the hidden Markov model (HMM) profile of the WRKY domain (PF03106) was used as a query to identify *WRKYs* using the program HMMER3.0 (http://hmmer.janelia.org). Finally, the sequences were compared with cDNA sequences of *WRKY* genes in PlantTFDB (http://planttfdb.cbi.pku.edu.cn) (Zhang et al. 2011) and the integrity of the WRKY domain was confirmed by SMART with default cut-off parameters (http://smart.embl-heidelberg.de/). After manually removing incorrect and overlapping predicted genes, 58 protein sequences were identified.

### Mapping *WRKY* genes on peach chromosomes

The locations of the *WRKY* genes on the chromosomes were obtained from Phytozome (http://phytozome.jgi.doe.gov/pz/portal.html). The *WRKY* genes were mapped to the chromosomes using Circos software (http://circos.ca/tutorials/lessons/).

### Phylogenetic analysis of *WRKY* genes in peach, *Arabidopsis*, and rice

The *WRKY* family sequences for *Arabidopsis* were retrieved from TAIR (http://www.arabidopsis.org/) and those for rice were retrieved from NCBI (http://www.ncbi.nlm.nih.gov). Peach sequences were identified by local BLASTP searches and HMM profiling as described above. The *WRKY* sequences were aligned using the ClustalX 2.1 (Larkin et al. 2007) with the default settings. Phylogenetic and molecular trees based on the protein sequences predicted from *WRKY* gene sequences were constructed using the neighbor-joining algorithm with the program MEGA6.0 (http://www.megasoftware.net/mega6/faq.html), with parameters set according to the JTT model. The reliability of the obtained trees was tested by conducting 1000 bootstrap sampling steps.

### Gene structure construction

The coding sequences (CDS) and genome sequences of *WRKY* genes in peach were downloaded from Phytozome (http://phytozome.jgi.doe.gov/pz/portal.html#!info?alias=Org_Ppersica_er). The gene structures were predicted using GSDS online (http://gsds.cbi.pku.edu.cn/).

### qRT-PCR analysis of *WRKY* gene expression during bud dormancy

Total RNA was extracted from buds with scales (500 mg) using the RNeasy Plus Mini Kit (Qiagen, Valencia, CA, USA) according to the manufacturer’s instructions. For the qRT-PCR, cDNA was synthesized using the PrimeScript RT reagent kit with gDNA Eraser (Takara, Dalian, China). The qRT-PCR was performed with SYBR Premix Ex Taq (Takara) following the manufacturer’s instructions. The sequences of gene primers (β-actin primer pair as an internal control)used for qRT-PCR (Table S1) were designed with BD software and synthesized by BGI (http://www.genomics.cn). The expression of the reference gene is not changed during all the development stages. We selected 36 *WRKY* genes for qRT-PCR analysis (Table S2). The thermal cycling conditions were as follows: 10 min at 95 °C for pre-denaturation, followed by 40 cycles of 15 s at 95 °C for denaturation and 60 s at 60 °C for annealing and extension. The specificity of the qRT-PCR was confirmed by the presence of a single peak in the melting curve and by size estimation of the amplified qRT-PCR product. To quantify cDNAs with amplification efficiencies, the comparative cycle threshold (CT) method (2^−ΔΔCT^) method was used. To further observe the changes of expression profiles between endodormancy and ecodormancy, the mean expression of each genes during endodormancy and ecodormancy was used. As shown in Fig. 5, endodormancy represents the mean expression of 16 and 31 October, 15 November, 1, 15 and 31 December and ecodormancy represents the mean expression of 15 and 25 January, and 15 February. Each reaction was repeated three times. Results are the average of three independent biological replicates.

## Results

### Identification of *WRKY* genes in peach

Members of the *WRKY*-gene family have been identified in many species, but not in peach until now. We used two approaches to identify members of the *WRKY* family in peach. First, all annotated proteins of peach were used as query sequences to perform BLASTP searches in the NCBI database. Then, the hidden Markov model (HMM) profile of the WRKY domain (PF03106) was used as a query to identify *WRKY* genes using the program HMMER3.0. After manually removing redundant sequences, the remaining genes were further analyzed to confirm the integrity of the WRKY domain using SMART with the default cut-off parameters. Finally, 58 non-redundant putative *WRKY* genes were identified in peach. The length, putative molecular weight, and theoretical isoelectric points of the WRKY TFs were analyzed in this study varied widely (Table 1). The length of the predicted WRKY TFs ranged from 170 to 751 amino acids, their putative molecular weights ranged from 19.6 to 82.2 kDa, and their theoretical isoelectric points ranged from 4.7 to 10.6.

**Table 1:** Information for *WRKY* gene family members in peach

Gene name	Locus name	Size (aa)	Molecular weight (KD)	PI
PpWRKYI				
*PpWRKY1*	Prupe.1G280700	517	56.2	7.5
*PpWRKY2*	Prupe.3G202000	486	52.8	6.6
*PpWRKY3*	Prupe.3G262100	547	59.7	7.5
*PpWRKY4*	Prupe.4G232600	586	64.0	6.4
*PpWRKY5*	Prupe.6G036300	740	80.0	6.1
*PpWRKY6*	Prupe.6G046900	584	64.1	7.1
*PpWRKY7*	Prupe.6G244300	475	51.7	8.8
*PpWRKY8*	Prupe.6G286000	535	59.3	7.3
*PpWRKY9*	Prupe.6G361300	751	82.2	6.2
*PpWRKY10*	Prupe.7G262600	533	58.8	5.4
PpWRKYIIa				
*PpWRKY11*	Prupe.1G393000	326	36.4	7.5
*PpWRKY12*	Prupe.1G393100	271	30.1	9.0
*PpWRKY13*	Prupe.3G098100	236	25.7	10.0
PpWRKYIIb				
*PpWRKY14*	Prupe.1G269200	533	58.1	7.8
*PpWRKY15*	Prupe.1G564300	561	62.1	4.8
*PpWRKY16*	Prupe.3G002300	567	62.0	6.8
*PpWRKY17*	Prupe.3G214800	651	70.9	6.7
*PpWRKY18*	Prupe.3G270800	481	52.7	7.5
*PpWRKY19*	Prupe.4G217900	513	56.0	6.6
*PpWRKY20*	Prupe.4G017600	564	62.5	7.3
*PpWRKY21*	Prupe.5G187800	646	69.5	7.4
PpWRKYIIc				
*PpWRKY22*	Prupe.1G114800	390	42.9	6.2
*PpWRKY23*	Prupe.1G223200	185	21.0	10.1
*PpWRKY24*	Prupe.1G283500	330	36.1	6.1
*PpWRKY25*	Prupe.1G407500	187	21.3	5.5
*PpWRKY26*	Prupe.2G133800	244	27.7	7.7
*PpWRKY27*	Prupe.2G177800	221	24.8	9.5
*PpWRKY28*	Prupe.2G231300	174	20.1	9.6
*PpWRKY29*	Prupe.3G008600	321	35.6	7.0
*PpWRKY30*	Prupe.3G174300	360	41.0	7.3
*PpWRKY31*	Prupe.3G308200	223	25.4	9.3
*PpWRKY32*	Prupe.4G075400	337	37.3	7.0
*PpWRKY33*	Prupe.5G148700	170	19.6	9.9
*PpWRKY34*	Prupe.6G168200	231	26.4	9.2
*PpWRKY35*	Prupe.6G169700	196	22.2	6.7
*PpWRKY36*	Prupe.6G257500	299	33.5	5.0
PpWRKYIId				
*PpWRKY37*	Prupe.1G071400	281	30.7	10.6
*PpWRKY38*	Prupe.1G431100	351	38.3	10.0
*PpWRKY39*	Prupe.1G459100	317	34.4	9.6
*PpWRKY40*	Prupe.5G074200	340	38.0	10.0
*PpWRKY41*	Prupe.6G230600	325	35.5	10.1
*PpWRKY42*	Prupe.6G345100	354	40.0	10.3
*PpWRKY43*	Prupe.8G230700	299	33.9	10.2
PpWRKYIIe				
*PpWRKY44*	Prupe.2G177400	357	39.2	7.6
*PpWRKY45*	Prupe.2G302500	402	44.9	5.4
*PpWRKY46*	Prupe.3G113300	277	30.0	6.8
*PpWRKY47*	Prupe.4G066400	283	30.9	5.8
*PpWRKY48*	Prupe.4G101100	504	54.4	6.4
*PpWRKY49*	Prupe.5G106700	283	31.8	4.7
*PpWRKY50*	Prupe.8G265900	258	29.3	5.2
PpWRKYIII				
*PpWRKY51*	Prupe.2G185100	358	39.6	4.7
*PpWRKY52*	Prupe.2G264900	348	37.8	6.7
*PpWRKY53*	Prupe.2G265000	323	36.3	6.0
*PpWRKY54*	Prupe.2G307400	349	38.1	5.2
*PpWRKY55*	Prupe.5G117000	326	36.5	6.2
*PpWRKY56*	Prupe.6G294900	350	39.2	6.5
*PpWRKY57*	Prupe.6G295000	335	38.0	6.2
*PpWRKY58*	Prupe.6G295100	286	32.2	7.6

### Phylogenetic analysis and classification of *WRKYs* in peach, *Arabidopsis,* and rice

**Fig. 1:**
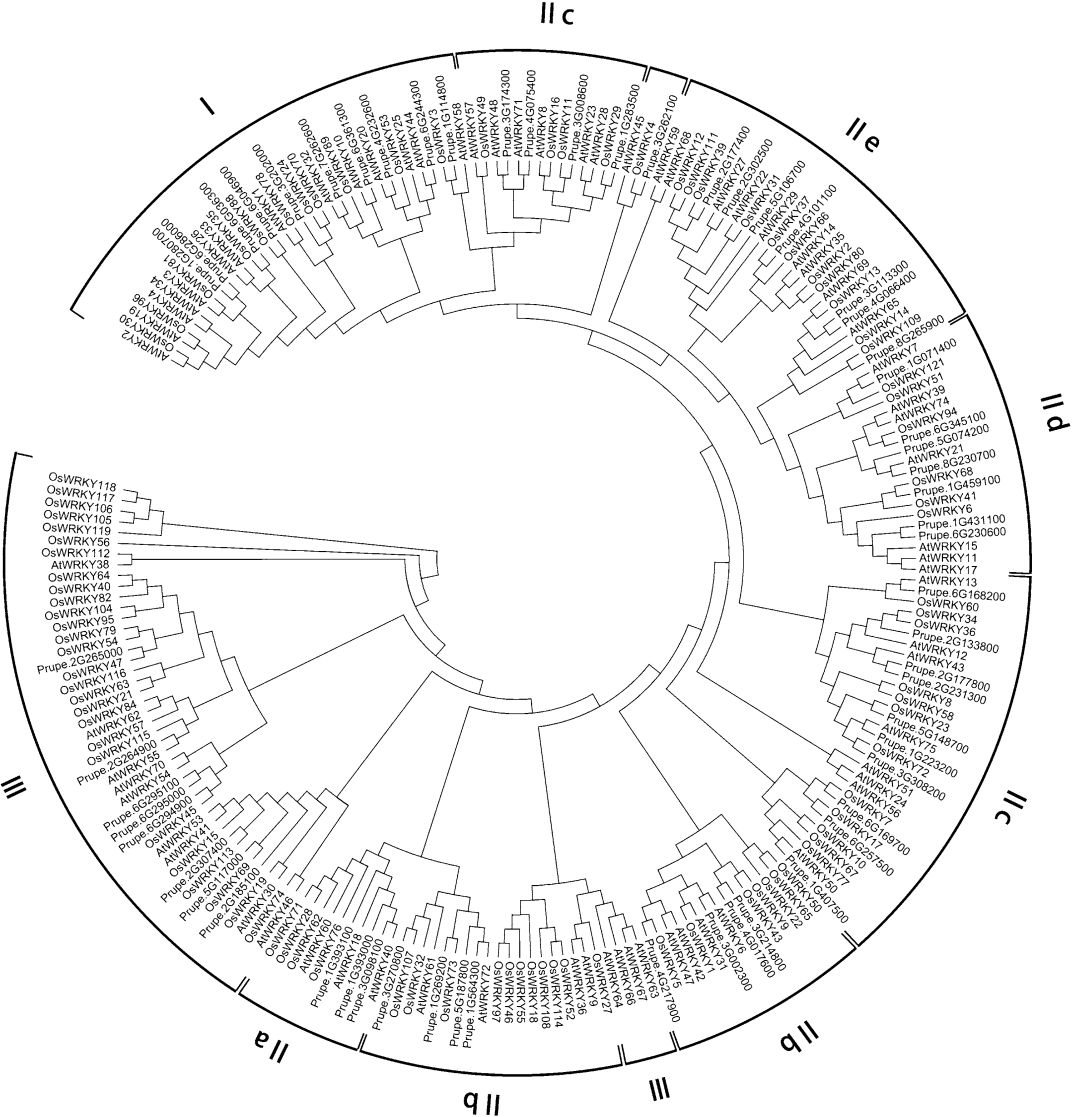
Phylogenetic tree of peach, rice, and *Arabidopsis* WRKY proteins. The 58 peach, 73 *Arabidopsis* and 94 rice protein sequences were aligned by ClustalW and the phylogenetic tree was constructed using MEGA6.0 by the neighbor-joining method with 1000 bootstrap replicates. WRKY proteins clustered into three main groups

To evaluate the phylogenetic relationships of the *WRKY* genes in peach and classify them within the established subfamilies, we analyzed 225 amino acid sequences containing the WRKY domain. These sequences consisted of 58 sequences from peach, 73 sequences from *Arabidopsis*, and 94 sequences from rice. The sequences of *Arabidopsis* WRKY proteins were downloaded from TAIR, those of peach were downloaded from Phytozome (http://phytozome.jgi.doe.gov/pz/portal.html#!info?alias=Org_Ppersica_er), and those of rice were downloaded from NCBI (http://www.ncbi.nlm.nih.gov). An unrooted phylogenetic tree (Fig. 1) was constructed using the neighbor-joining method with MEGA6.0 (http://www.megasoftware.net/history.php) (Tamura et al. 2013). In the phylogenetic tree, the *WRKY* genes in peach were divided into three main groups: PpWRKY I, II, and III, according to their predicted WRKY domains and zinc finger structures. There were 10 WRKY TFs in group I, 40 PpWRKYs in group II, and 8 PpWRKYs in group III. Members of group I contained two conserved domains and one C_2_H_2_ zinc finger motif, members of group II contained one conserved domain, and members of group III harbored the other conserved domain. The difference between groups II and III was the type of zinc finger motif; group II members had the same zinc finger motif as that in group I, while group III members contained the C_2_HC zinc finger motif. Group II contained five subgroups (PpWRKY IIa, b, c, d, and e, containing 3, 8, 15, 7, and 7 WRKY TFs, respectively). As shown in the phylogenetic tree, all the WRKY TFs from different species were clustered into three groups, and the WRKY TFs of different species in the same group were more similar than those from the same species in different groups. A previous study showed that WRKY TFs from various species harbor different variants of the WRKY domain (WRKYGQK), such as WRKYGKK, WRKYGSK, and WRKYGEK (Zhang and Wang 2005). In our study, we detected the WRKYGKK variant in Prupe.1G407500 and Prupe.6G169700 in group IIc. This was the only variant in peach (Yang et al. 2015). Interestingly, members of group IIb contained another highly conserved tetrapeptide sequence, the LDLT sequence (Supplementary 3).

### Distribution of *WRKY* genes on peach chromosomes

**Fig. 2:**
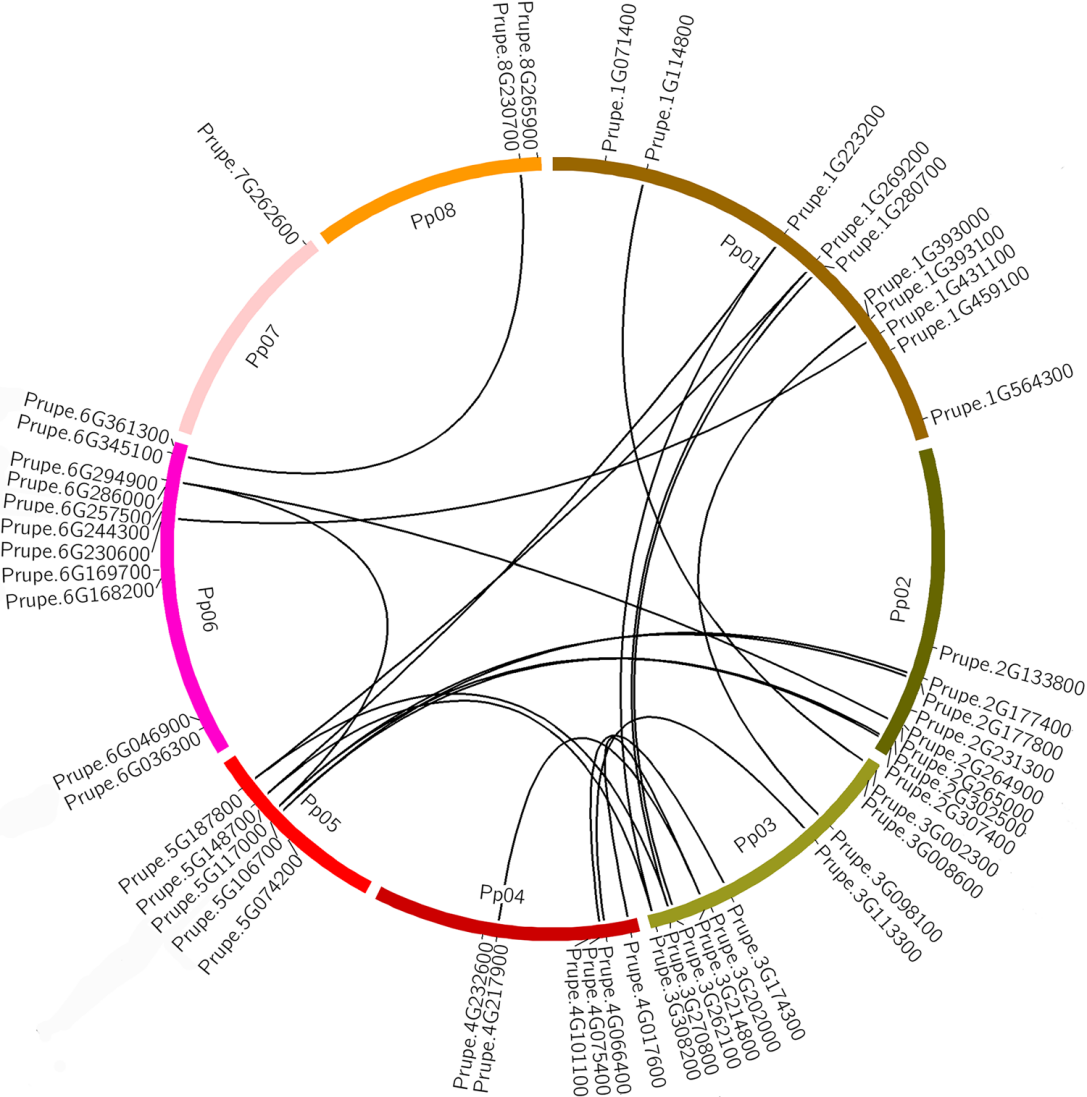
Distribution of 58 *WRKY* genes on eight peach chromosomes. Colinear *WRKY* genes which are paralogs formed by a duplication event are shown

Figure 2 shows the distribution of the 58 *WRKY* genes on peach chromosomes. As shown in the figure, the *WRKY* genes were unevenly distributed throughout all eight peach chromosomes, and the number on each chromosome was not related to its length. Chromosome 6 had the most *WRKY* genes (13 genes, or 22.4 % of the total) followed by Chr1 (12 genes), while Chr7 had the least (one *WRKY* gene). Nine *WRKY* genes were located on Chr2, 10 on Chr3, 6 on Chr4, 5 on Chr5, and 2 on Chr8. The nomenclature of the *PpWRKYs* was established from the exact position of the *WRKY* genes on peach chromosomes 1 to 8, from top to bottom, and from their classifications.

Previous reports have indicated that gene duplication and positive selection have significantly contributed to the expansion of gene families and the diversification of protein functions (Wei et al. 2012). To understand the role of gene duplication in the expansion of the *WRKY* gene family, we analyzed the tandem and segmental duplications of this gene family. The results revealed 30 colinear *WRKY* genes and nine tandem *WRKY* genes. The collinear genes are consecutive genes along a genomic region that by a duplication event have paralogs in the same consecutive order in another genomic region. Interestingly, all the colinear *WRKY* genes within the syntenic regions belonged to the same group. Only two sets of triplicate *WRKY* genes were identified (Prupe.1G223200/Prupe.5G148700/Prupe.3G308200 and Prupe.5G187800/Prupe.1G269200/Prupe.3G270800). It is interesting to find that the two sets of triplicate *WRKY* genes are located on triplicated regions in the peach genome (Verde et al. 2013). Surprisingly, all chromosomes except Chr7 had *WRKY* genes located in the colinear duplicated regions.

### Structure of *WRKY* genes

**Fig. 3:**
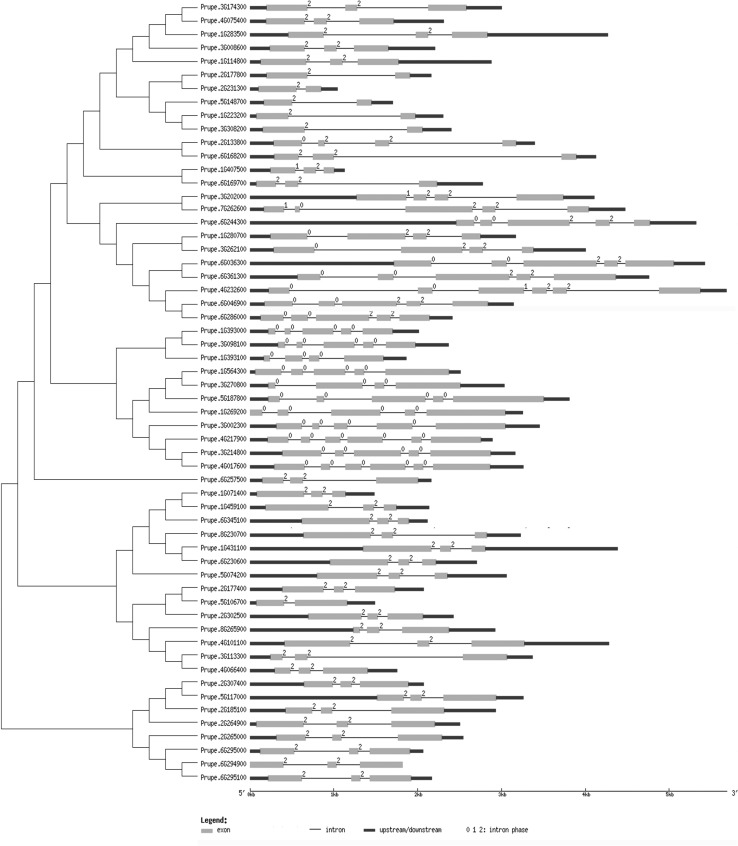
Phylogenetic analysis and structures of *WRKY* genes in peach. Phylogenetic tree was constructed by MEGA6.0 with the neighbor-joining method based on alignments of complete predicted protein sequences of *WRKY* genes. In gene structure diagram, *green boxes* and *lines* represent exons and introns, respectively

Considering that gene structure is a typical imprint of evolution within a gene family, we analyzed the *WRKY* genes in peach using tools at the GSDS website (Fig. 3). Interestingly, all of the *WRKY* genes in peach had one or more (up to five) introns, so each *PpWRKY* sequence was divided into many segments by introns. The genes in each group showed similar structures and similar intron phases. The numbers and phases of introns were more conserved in groups II and III than in group I. Members of group I contained phase 0, phase 1, and phase 2 introns. Compared with Prupe.3G202000, Prupe.4G232600 had two phase 0 introns before a phase 1 and two phase 2 introns. We inferred that there was no significant impact on mRNA level, but further research is required to determine whether the two phase 0 introns played a role in processing of the primary transcripts.

### Measurement of bud dormancy status and evaluation of stage-specific expression of *WRKY* genes during dormancy by qRT-PCR

**Fig. 4:**
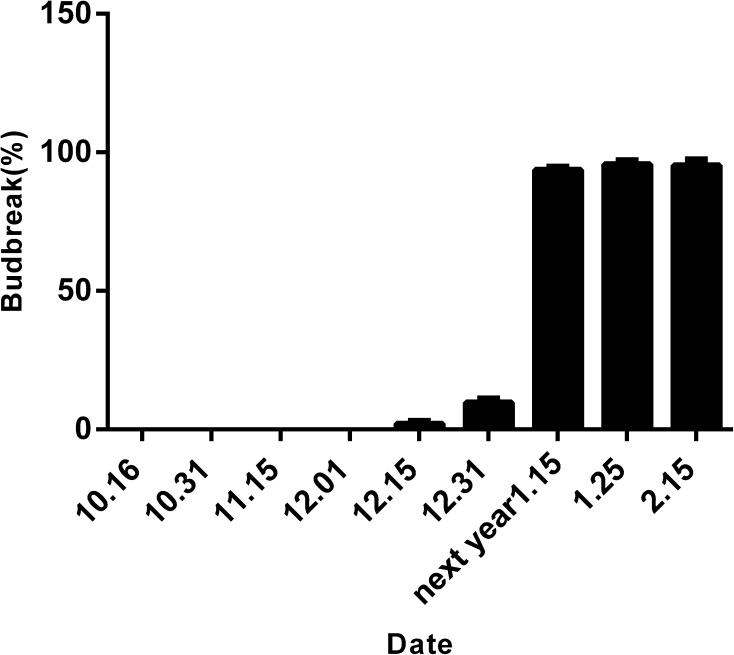
Measurement of bud dormancy, showing frequency of bud sprouting from 15 October until 15 February in the following year

To measure the transcript profiles of *WRKY* genes during dormancy in peach, the dormancy status of buds was defined for shoots of 5-year-old ‘*Prunus persica* L. cv Zhong You Tao 4’ peach trees collected on nine dates. As shown in Fig. 4, the first buds sprouted on 15 December, there was a marked increase in sprouting from early January, and then sprouting reached almost 100 %. Thus, the buds sampled from 15 October to 1 January represent endodormant buds, and those sampled from 15 January to 15 February represent ecodormant buds.

**Fig. 5:**
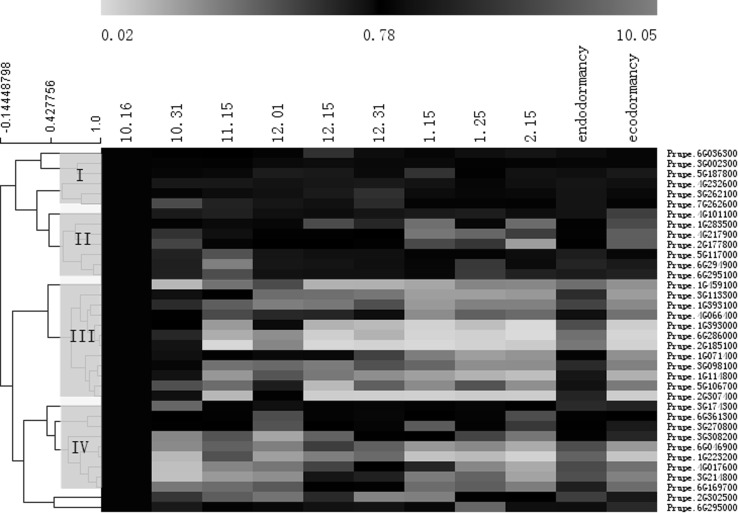
Relative expression profiles of *WRKY* genes during bud dormancy. Analyses of gene expression in buds were performed by qRT-PCR. Expression levels were normalized against that of Prupe.3G205200

*WRKY* genes are thought to be involved in regulating dormancy. To identify dormancy-related *WRKY* genes, the transcript profiles of *WRKY* genes at different stages of bud dormancy were analyzed by qRT-PCR. The 36 *WRKY* genes were classified into four gene expression groups using MeV software (Eisen et al. 1998) according to the chronological stages of bud dormancy (Fig. 5). Considering the inherent errors in the experimental set-up, we chose a three-fold change in expression as the definition of a dormancy-related *WRKY* gene. From the mean expression level, the expressions of Prupe.6G286000, Prupe.1G393000, Prupe.1G114800, Prupe.1G071400, Prupe.2G185100 and Prupe.2G307400 were up-regulated in endodormancy and down-regulated in ecodormancy, whereas during endodormancy the expressions were down-regulated concomitantly with endodormancy release. The other genes including Prupe.1G280700, Prupe.3G202000 Prupe.4G232600, Prupe.6G036300 had no obviously changes during dormancy.

## Discussion

With the development of genome sequencing projects, genome-wide analysis of the *WRKY* gene family has been reported for various species, such as *Arabidopsis* (Wu et al. 2005), soybean (Ülker and Somssich 2004), *Carica papaya* (Pan and Jiang 2014), cotton (Dou et al. 2014), *Gossypium raimondii* (Cai et al. 2014), grape (Wang et al. 2014), *Aegilops tauschii* (Ma et al. 2015), *Cucumis sativus* (Jian et al. 2011), *Brachypodium distachyon* (Feng et al. 2014), *Solanum lycopersicum* (Chen et al. 2015), *Gossypium* (Ding et al. 2015), and *Camellia sinensis* (Wu et al. 2015). However, the *WRKY* genes in *Prunus persica* had not been characterized until now. Therefore, the identification of *WRKY* genes in the peach genome and analyses of their expression patterns are important topics.

**Fig. 6:**
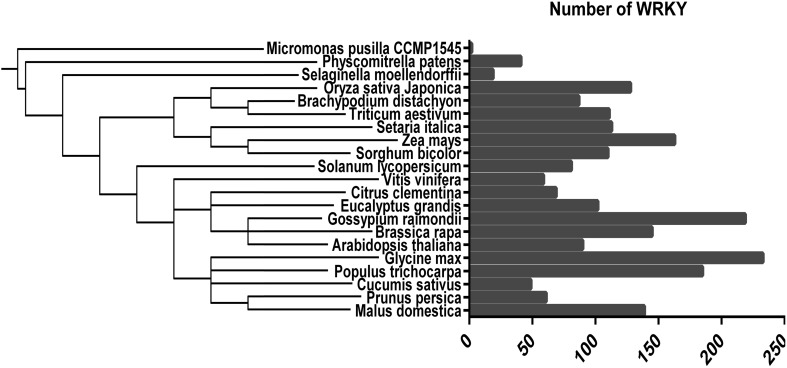
Distribution of WRKY transcription factors in different species (Letunic and Bork 2007, 2011)

Previous studies have shown that the bHLH family is the largest TF family in peach, while the *WRKY* gene family is the eighth-largest. To investigate the evolution of *WRKY* genes in plants, we compared 21 diverse plant species including those from the Chlorophyta to Embryophyta subkingdoms and determined how many *WRKY* genes were present in each species (Fig. 6). The number of *WRKY* genes in the various species ranged from 2 to 233. Embryophyte species had more *WRKY* genes than Chlorophyte species, suggesting that *WRKY* genes have played a vital role during evolution. Within the Rosaceae, *Malus domestica* had more *WRKY* genes than did peach, possibly because of the two genome replication events that occurred in *M. domestica* during evolution.

### Duplication of *WRKY* genes in peach

In peach, all seven paleosets of paralogs can be detected in fragmentary triplicated blocks. Peach has not undergone a recent whole genome duplication (Verde et al. 2013). The WRKY transcription factors have a long history and ancient origin in eukaryotes, which originally had one *WRKY* gene (Zhang and Wang 2005). The results of our analyses indicate that the gene duplication in peach and the distribution of the 58 *WRKY* genes on peach chromosomes are non-uniform. Gene duplication is the main driving force in evolution, and takes several forms; tandem duplication, segmental duplication, and whole genome duplication (Xu et al. 2012). The homologous genes (12 pairs of *WRKY* genes plus two sets of triplets) in peach were identified in the same homologous blocks, and the two sets of triplets were consistent with the fragmentary triplicated blocks. Compared with peach, *Arabidopsis* has 25.9 % more *WRKY* genes, and rice has 62.1 % more. The smaller number of *WRKY* genes in peach may be because unlike *Arabidopsis* and rice, peach has not undergone a recent whole-genome duplication after the differentiation of eudicots and monocots. There are tandem duplicates in the peach *WRKY* gene family; for some pairs, the two genes may have different functions. Alternatively, two functional genes may be required when a large transcript abundance is necessary for specific responses at specific times.

### Colinear orthologs of *WRKY* genes among peach, rice, *Arabidopsis*

Comparisons of genomic data between well-characterized and less-studied taxa can allow us to infer details of genome structure, function, and evolution of less-studied species based on knowledge gained from model species. Therefore, comparative genomic analysis is a relatively rapid and effective method for evaluating less-studied taxa (Lyons et al. 2008). The functions of the *WRKY* gene family have been widely studied in model plants such as *Arabidopsis* and rice. In theory, we can estimate the potential functions of *WRKY*s in peach by comparisons with their well-characterized homologs in *Arabidopsis* and rice. In the phylogenetic tree, many homologous genes from *Arabidopsis*, rice, peach with common conserved motifs clustered into the same clade.

In a previous study, *ABF1* and *ABF2*, two WRKY TFs from wild oat were found to be GA-inducible and ABA-repressible, like their homologs in rice and barley (Rushton et al. 1995). In rice, *OsWRKY51* and *OsWRKY71* were shown to function synergistically by forming a heterotetramer to control the production of α-amylase. *OsWRKY51/OsWRKY71* not only antagonized GAMYB but also prevented it forming a complex with other proteins, thus repressing ABA-induced dormancy (Xie et al. 2005, 2006; Zhang et al. 2004). In our BLAST searches, we identified the colinear orthologs of *OsWRKY51*/*OsWRKY71* in peach (Prupe.1G071400 and Prupe.3G098100). These genes showed almost identical expression profiles; therefore, we speculated that they may have the same functions in dormancy. Further studies are required to determine whether the peach orthologs form a tetramer, like their orthologs in rice.

### Expression profiles among paralogs in peach *WRKY* gene family

Some genes and their paralogs play redundant roles *in planta*, such as *AtWRKY18*, *AtWRKY40*, *AtWRKY60* (Xu et al. 2006), *AtWRKY54,* and *AtWRKY70* (Besseau et al. 2012; Li et al. 2013). However, some paralogs, such as *AtWRKY4* and *AtWRKY3,* have different functions. Previous studies have shown that the expression level of *AtWRKY4* but not *AtWRKY3* increased in response to *B. cinerea* infection. In our study, we analyzed the expression profiles of paralog pairs or triplets (Prupe.1G393000 and Prupe.1G393100; Prupe.6G168200 and Prupe.6G169700; Prupe.2G264900 and Prupe.2G265000; Prupe.6G294900, Prupe.6G295000, and Prupe.6G295100) in the peach *WRKY* gene family. Prupe.6G168200 and Prupe.6G169700 showed the same expression profiles, while the others had non-identical profiles and showed different expression levels during dormancy. We inferred that differences in expression between and among paralogs may be related to sequences outside the conserved motif. Our results suggested that some paralogs in peach are redundant, while others have diverse functional roles.

### *WRKY* gene family may be involved in dormancy

WRKY transcription factors have been shown participate in many plant processes such as biotic stress, abiotic stress, seed development, seed dormancy and germination, senescence, and development (Craig and Ling 2014). According to QTLs in peach, G1, G4, G6/8 and G7 were detected associated with controlling seed dormancy (Blaker et al. 2013). Romeu et al. (2014) also identified that QTLs related to bud dormancy in peach mainly mapped to LG1which closes to the *evergrowing* locus. Based on these, we performed analysis which compares the genomic sites for the QTLs with reference to the location of the relevant *WRKY* genes on the peach genome. Interestingly, we found that some *WRKY* genes including Prupe.1G071400 mapped to LG1, Prupe.7G262600 mapped to G7 and Prupe.4G232600 mapped to G4. All these demonstrated the *WRKY* genes may be involved in dormancy. A previous study showed that 15 *WRKY* genes in grape had identical expression patterns under cold treatment (Wang et al. 2014). Another study showed that Prupe.1G071400 might play roles in the early responses to abiotic stress, in acquiring resistance, and in controlling dormancy. In our study, the mean expression of Prupe.1G071400 was at relatively high level in endodormancy and at lower level in ecodormancy, further indicating that this gene may participate in regulating dormancy. Similarly, other *WRKY* genes (Prupe.6G286000, Prupe.1G393000, Prupe.1G114800, Prupe.2G185100 and Prupe.2G307400) were detected specifically in endodormant buds compared with ecodormant buds, suggesting that they may be related to dormancy. Along with the changes in the process of endodormancy, most of the studied genes including Prupe.1G393000, Prupe.1G 071400 reduced their expression at December 15 previous to dormancy release which further illustrates *WRKY* genes may be involved in endodormancy. Considering that internal factors inhibit the growth of endodormant buds even in favorable conditions (Lang et al. 1987), we inferred that *WRKY* genes may act as internal factors controlling endodormancy. However, transgenic studies are required to evaluate the functions of *WRKYs*.

In conclusion, the peach genome contains 58 *WRKY* genes that are unevenly distributed on all eight chromosomes. The phylogenetic, gene structure and chromosomal location analyses have provided complete information for this gene family in peach. Segmental duplication has played a vital role in the expansion of the *WRKY* gene family in peach. The expression profiles of *WRKY* genes during dormancy demonstrated that some of them may be involved in dormancy. In summary, the results of this study provide the foundation for further studies on the roles of *WRKY* genes in bud dormancy and will be useful for further research on the evolutionary history of *WRKY* genes in eukaryotes.

## Electronic supplementary material

Below is the link to the electronic supplementary material.

Supplementary material 1 (TIFF 11 kb)

Supplementary material 2 (DOC 53 kb)

Supplementary material 3 (DOCX 16 kb)
