# Supplementary figures and images for: Genome-wide identification of WRKY family genes in peach and analysis of WRKY expression during bud dormancy
Source: Mol Genet Genomics. 2016 Mar 7;291:1319–32. doi: 10.1007/s00438-016-1171-6 (PMC4875958; doi:10.1007/s00438-016-1171-6)

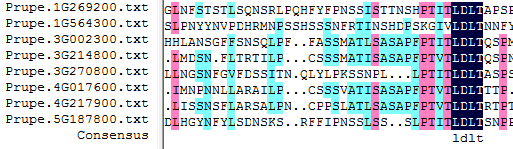

Supplement: Supplementary file 1 — Supplementary material 1 (TIFF 11 kb) [file 438_2016_1171_MOESM1_ESM.tif]
