# Supplementary material for: Genome-wide identification of WRKY family genes in peach and analysis of WRKY expression during bud dormancy
Source: Mol Genet Genomics. 2016 Mar 7;291:1319–32. doi: 10.1007/s00438-016-1171-6 (PMC4875958; doi:10.1007/s00438-016-1171-6)
Supplement: Supplementary file 2 — Supplementary material 2 (DOC 53 kb) [file 438_2016_1171_MOESM2_ESM.doc]

| **Accession number** | **Forward Primer(5'-3')** | **Reverse Primer(5'-3')** |
| --- | --- | --- |
| Prupe.6G036300 | GCAACCAATCCTCTTCTATG | ATTCATCTCCTTCACCATCA |
| Prupe.3G214800 | GATTGGCTGCTAATAATGCT | AACTCTGTGATGGCTGAT |
| Prupe.6G046900 | CAGGGAAGAGTATGGTGAA | GATTGTTGGTGGTTGTAGTT |
| Prupe.4G017600 | GGCTTCTTCCTCAACAAG | TCCACAACCACACGATTA |
| Prupe.3G262100 | GCTGTCATCACTACATACG | TACTGGTCTCTGGTCATTG |
| Prupe.6G286000 | GCCTTGCCTAATCATTCG | TCCATAACTTCCTTCACTCT |
| Prupe.7G262600 | TGTCAGAACTATCACCAACT | AATGGATTGCGTAACAGAAG |
| Prupe.4G101100 | TCATCACCACCATCTTCAT | CAGACACCGCCTTACTAA |
| Prupe.1G114800 | CGATGATGATGGTGATGATG | ACGACGAAGAGATTGAAGA |
| Prupe.1G283500 | GCTCAAGAATCTGGGTTTG | CATCATCATCCTCATCATCAC |
| Prupe.1G393000 | CTCTGATACACGCCTGAT | TGTGGTTATGTTCTCCTTCA |
| Prupe.1G459100 | TCAGAAGGCTATACAGGAAG | ATACGAGGGATGAGAAGAAG |
| Prupe.2G177800 | GAGTGCCGATGATATTCTTG | AATGCTCGTGTCCTTAGAT |
| Prupe.1G223200 | GCTTCTTCTTCCTTGTCATT | CCTAACCTCACAGCAGTT |
| Prupe.3G308200 | CCTTGCTCCTCTTCTACAT | TGGTTTCCTGCTGATGAT |
| Prupe.6G295000 | GGCGGCTGTTATTAAGTC | TTGAGTGCTTGGAATTAAGG |
| Prupe.4G217900 | GCAGAAGACCGAACAATAC | TTGAGCCTGATAGTAGCATT |
| Prupe.5G106700 | AGTCATTCTTGCCTCCTC | CTTCTGCTCTCACCTCTT |
| Prupe.6G295100 | GGCGACATTAAGCAAGAC | TTAGGAGTGATGAGGACATAG |
| Prupe.2G185100 | GGCTATTCTCCCACATTTG | GAGTTGGTCACTGAAGTTG |
| Prupe.2G307400 | GCTTTCCATGCTCAATTCT | TTTGTTGTCCTGGTCCTT |
| Prupe.5G117000 | TTCATCAGACACCAGACAA | GTAAGCCGACACAAGAGA |
| Prupe.1G071400 | AAGGTTAGGAGGACGATTAG | TCACGCTGCTACACTTAT |
| Prupe.2G177800 | AGTCAATCACGATGCATGAGAA | TAGACTTCTGGTGCTGAC |
| Prupe.2G185100 | GGCTATTCTCCCACATTTG | GAGTTGGTCACTGAAGTTG |
| **Accession number** | **Forward Primer(5'-3')** | **Reverse Primer(5'-3')** |
| Prupe.6G294900 | AGTGGTCAACTTGGTAGG | AACTCCTTCATCTCCTTCC |
| Prupe.5G106700 | AGTCATTCTTGCCTCCTC | CTTCTGCTCTCACCTCTT |
| Prupe.1G459100 | TCAGAAGGCTATACAGGAAG | ATACGAGGGATGAGAAGAAG |
| Prupe.3G205200(ACTIN) | GTTATTCTTCATCGGCGTCTTCG | CTTCACCATTCCAGTTCCATTGTC |
