# Supplementary material for: Genome-wide identification of WRKY family genes in peach and analysis of WRKY expression during bud dormancy
Source: Mol Genet Genomics. 2016 Mar 7;291:1319–32. doi: 10.1007/s00438-016-1171-6 (PMC4875958; doi:10.1007/s00438-016-1171-6)
Supplement: Supplementary file 3 — Supplementary material 3 (DOCX 16 kb) [file 438_2016_1171_MOESM3_ESM.docx]

| Group | Gene identifier |
| --- | --- |
| GroupⅠ | Prupe.3G262100, Prupe.4G232600, Prupe.6G036300, Prupe.6G046900, Prupe.6G286000, Prupe.6G361300, Prupe.7G262600 |
| GroupⅡa | Prupe.1G393000, Prupe.1G393100, Prupe.3G098100 |
| GroupⅡb | Prupe.3G002300, Prupe.3G214800, Prupe.3G270800, Prupe.4G217900, Prupe.4G017600, Prupe.5G187800 |
| GroupⅡc | Prupe.1G114800, Prupe.1G223200, Prupe.1G283500, Prupe.2G177800, Prupe.3G174300, Prupe.3G308200, Prupe.6G169700 |
| GroupⅡd | Prupe.1G459100 |
| GroupⅡe | Prupe.1G071400, Prupe.2G302500, Prupe.3G113300, Prupe.4G066400, Prupe.4G101100, Prupe.5G106700 |
| GroupⅢ | Prupe.2G185100, Prupe.2G307400, Prupe.5G117000, Prupe.6G294900, Prupe.6G295000, Prupe.6G295100 |
